# Biochar influences nitrogen and phosphorus dynamics in two texturally different soils

**DOI:** 10.1038/s41598-024-55527-2

**Published:** 2024-03-19

**Authors:** Rajeev Kumar Gupta, Monika Vashisht, R. K. Naresh, Nitish Dhingra, Mehra S. Sidhu, P. K. Singh, Neeraj Rani, Nadhir Al-Ansari, Abed Alataway, Ahmed Z. Dewidar, Mohamed A. Mattar

**Affiliations:** 1 School of Agriculture, Lovely Professional University, Jalandhar, 144001 Punjab India; 2 Department of Agronomy, Sardar Vallabhbhai Patel University of Agriculture & Technology, Meerut, 250110 U.P. India; 3 Electron Microscopy & Nanoscience Laboratory, Department of Soil Science, Punjab Agricultural University, Ludhiana, 141004 Punjab India; 4 Director Extension, Sardar Vallabhbhai Patel University of Agriculture & Technology, Meerut, 250110 U.P. India; 5 School of Organic Farming, Punjab Agricultural University, Ludhiana, 141004 India; 6 Department of Civil, Environmental and Natural Resources Engineering, Lulea University of Technology, 97187 Lulea, Sweden; 7 Prince Sultan Bin Abdulaziz International Prize for Water Chair, Water and Desert Research, Prince Sultan Institute for Environmental, King Saud University, P.O. Box 2454, 11451 Riyadh, Saudi Arabia; 8 Department of Agricultural Engineering, College of Food and Agriculture Sciences, King Saud University, P.O. Box 2460, 11451 Riyadh, Saudi Arabia

**Keywords:** Acacia wood biochar, Incubation, Nitrogen, Phosphorus, Rice straw biochar, Soil, Transformations, Agroecology, Environmental sciences

## Abstract

Nitrogen (N) and phosphorus (P) are vital for crop growth. However, most agricultural systems have limited inherent ability to supply N and P to crops. Biochars (BCs) are strongly advocated in agrosystems and are known to improve the availability of N and P in crops through different chemical transformations. Herein, a soil-biochar incubation experiment was carried out to investigate the transformations of N and P in two different textured soils, namely clay loam and loamy sand, on mixing with rice straw biochar (RSB) and acacia wood biochar (ACB) at each level (0, 0.5, and 1.0% w/w). Ammonium N (NH_4_-N) decreased continuously with the increasing incubation period. The ammonium N content disappeared rapidly in both the soils incubated with biochars compared to the unamended soil. RSB increased the nitrate N (NO_3_–N) content significantly compared to ACB for the entire study period in both texturally divergent soils. The nitrate N content increased with the enhanced biochar addition rate in clay loam soil until 15 days after incubation; however, it was reduced for the biochar addition rate of 1% compared to 0.5% at 30 and 60 days after incubation in loamy sand soil. With ACB, the net increase in nitrate N content with the biochar addition rate of 1% remained higher than the 0.5% rate for 60 days in clay loam and 30 days in loamy sand soil. The phosphorus content remained consistently higher in both the soils amended with two types of biochars till the completion of the experiment.

## Introduction

Nitrogen and phosphorous are indispensable for plant growth. The supply of significant amounts of nitrogen and phosphorus in farmlands is a common practice by farmers to augment the growth of crops, responsible for non-point source pollution and bringing an indisputable soil environment^1^. Despite the excess of these nutrients, most agricultural systems cannot supply nitrogen and phosphorus to crops^2^. Sustainable approaches are in demand to utilize the excessive nutrient stores of soils for better assimilation in plant biota. Biochar application for augmenting soil fertility is gathering momentum globally to achieve desired agronomic outcomes^3^^,^^4^. Biochar is a carbon-rich material produced by pyrolysis under minimal or zero oxygen supply for its application to the soil and is recognised as a sustainable methodology to improve soil properties and nutrient availability^5^. Its production and application to soils have been in advocation for a long as an effective method for recycling biomass along with benefiting soil carbon (C) sequestration, soil moisture and nutrient retention, and alleviating nutrient leaching^6^. Biochars, diversly heterogeneous in their form and reactivity in soil, are reported to affect soil N and P dynamics. Various biochar studies have focused on row crop agricultural systems associated with external nutrient inputs^7,8^, along with the effect of variously prepared biochars on nitrogen (N) and phosphorus (P) transformations in soils. Adsorption of phosphate and ammonium N (NH_4_-N) ions on biochars eventually leads to less N availability for nitrification^9,10^. Some reports state that biochar addition increased net nitrification rates in temperate and boreal forest soils^11^ as it stimulates or inhibits soil microbial activity.

P application and availability in agricultural soils is a matter of immense pertinence to maintaining high crop productivity. Alteration in the concentration of P on biochar-amended soil is often used as a possible explanation for biochar effects on improved crop growth^12,13^, which is reportedly due to biochar anion exchange capacity, different adsorption effects^14^ or by influencing the activity of cations that interacts with soil P. The study^15^ found that the addition of biochar reduced P leaching from manured soil due to the sorption of both orthophosphate and organic P by biochar. The information available in the literature on the effects of biochar on nutrient transformation in soil needs to be more consistent. The mechanisms and processes of P reaction dynamics in biochar-amended soils need further investigation^16,17^.

Though various reports are there on the effect of adding biochars on N and P, the information is limited to N and P transformations in some agricultural soil types. Moreover, the effects of biochar on N and P transformations depend on the type of biochar and soil^2^. Laboratory incubation studies of biochar application on N and P transformations in soils (varying in texture) is a pertinent concept that needs a critical examination. In this contextthe effect of rice straw biochar (RSB) and acacia wood biochar (ACB) are put on incubation trails on clay loam and loamy sand soils to study the transformation pattern of nitrogen and phosphorous. Such fundamental knowledge is crucial for developing guidelines for the selection and appropriate use of biochar and evaluating the potential sustainable use of biochar in agricultural soils.

## Materials and methods

### Biochar production

Rice (*Oryza sativa L.*) straw was collected from research farms at Punjab Agricultural University, Ludhiana, Punjab. The straw was air-dried at room temperature and ground to pass through a 10-mesh sieve. The biochar was prepared as per the previously reported methodology^18^ at a temperature of 380 °C for an incubation time of 3 h.

The ACB was prepared using the procedure outlined in Gupta et al.^19^, rationally, a pyramid-shaped structure (earth kiln) of wood is piled for its preparation. Vents are provided from the top to downwards to allow the combustion products to escape. When smoke production is ceased, the cooling process is initiated by pouring a layer of moist earth on this structure. The cooling process is completed in two to three days. Then, the biochar is separated from the surrounding carbonized portions before removing the earth, finely grounded and analyzed for basic properties.

### Laboratory incubation study

Effects of biochar on nitrogen and phosphorus transformations were determined through a laboratory incubation study with loamy sand and clay loam soils. Bulk soil samples (0–15 cm) representing clay loam and loamy sand were obtained from two distinct fields 1.5 km apart at the Punjab Agricultural University's experimental farm in Ludhiana, Punjab, India. The samples were further air-dried, passed through a 0.5 mm sieve and analyzed for initial physical and chemical properties using standard analytical methods.

### Effect of biochar type and application rates on nitrogen transformation in soils

Treatments included RSB and ACB, each mixed with soil at three rates (0, 0.5 and 1% on a w/w basis). Nitrogen as urea was applied at 0 and 100 mg Nkg^−1^. The treated pots followed a completely randomized design (CRD) in three factorial combinations: (a) type of biochar, (b) rates of biochar, and (c) two N-rates. Each treatment was replicated thrice. A known weight of biochar was thoroughly mixed with 750 g of soil (on a dry weight basis). To one set of treated soil, 100 mg N kg^−1^ was applied in solution form, while the other set was kept as no-N control. In total, there were 12 treatments for each soil type. All the treatments were thoroughly mixed with soil, and distilled water was adjusted to 75% of field capacity (16% for clay loam and 11% for loamy sand; dry weight basis). Thereafter, treated soil samples were transferred into plastic containers (10 cm internal diameter). Sealed containers were arranged in CRD in the incubator at 25 °C for 60 days. Soil sub-samples from each container were drawn at 1, 3, 5, 7, 15, 30, and 60 days after incubation to determine soil wetness, ammonium-N and nitrate–N contents.

### Effect of biochar type and application rates on phosphorus transformation in soils

The two biochar types and similar application rates were used. The P as KH_2_PO_4_ was applied at 0 and 30 mg P kg^−1^. The treated pots were placed in triplicate in a CRD with three factorial combinations: (a) type of biochar, (b) biochar rates, and (c) two P rates. Soil subsamples from each container were drawn at 1, 3, 5, 7, 15, 30, and 60 days after incubation to determine soil moisture and Olsen-P contents.

### Analytical procedures

#### Biochar analysis

The pH, electrical conductivity (EC), total carbon (C), nitrogen (N), ammonium N (NH_4_–N), nitrate N (NO_3_–N), phosphorus (P), and potassium (K) concentrations of biochar samples were determined. Total C and N contents were determined by a CHN analyser (Elementar Vario EL). The pH and EC of the biochar were determined in the solution having a 1:5 biochar to water ratio. The NH_4_-N and NO_3_-N contents in the 2 M KCl extracts were calculated as described by Ref.^20^. Total P and K contents in di-acid digests were determined on Inductively Coupled Argon Plasma (ICAP). The chemical composition of the biochars is illustrated in Table 1.

**Table 1:** Chemical composition of rice straw biochar and acacia biochar.

Determinant	Rice straw biochar (RSB)	Acacia biochar (ACB)
pH (1: 5)	9.09	7.26
EC (dS m^−1^)	0.85	0.13
Total N (%)	0.52	0.20
Total K (%)	9.2	0.85
Total P (%)	0.37	0.11
Total C (%)	30.1	48.0
C: N ratio	58:1	240:1

#### Soil analysis

The International Pipette Method described by Ref.^21^ was used to determine the particle size distribution of soil samples. The USDA textural triangle was then used to determine the textural class. Soil pH and EC were measured using a glass electrode pH meter and a conductivity meter in a 1:2 soil-to-water ratio. Wet digestion was used to calculate organic carbon^22^. Total N was determined by digesting soil samples with concentrated H_2_SO_4_ digests, as described in Ref.^23^. The available P was determined in 0.5M NaHCO_3_ soil extracts as outlined by Ref.^24^. The amount of available K in 1N NH_4_OAc extracts was determined using a flame photometer^25^. A MicroKjeldahl distillation method was used to detect inorganic N (NH_4_−N and NO_3_−N) in 2M KCl extracts. The selected properties of the two soils used in the incubation study are given in Table 2. The pH of clay loam and sandy loam soil was 7.62 and 6.95, respectively, and both soils were non-saline. Clay loam soil contained 6.0 g kg^−1^ of organic carbon and was high in both available P (34.2 kg ha^−1^) and K (336 kg ha^−1^). In contrast, loamy sand soil tested low in organic carbon (2.93 g kg^−1^), medium in available P (13.6 kg ha^−1^) and available K (275 kg ha^−1^).

**Table 2:** The initial soil properties of the experimental sites.

Soil texture	Soil I	Soil II
	Clay loam	Loamy sand
pH (1:2)	7.62	6.95
EC (dS m^−1^)	0.343	0.165
Organic C (g kg^−1^)	6.00	2.93
NH_4_-N (mg kg^−1^)	34.1	9.8
NO_3_-N (mg kg^−1^)	6.2	26.2
Olsen-P (mg kg^−1^)	15.3	60.1
NH_4_OAc extractable K (mg kg^−1^)	150.0	123.0

### Statistical analysis

Analysis of variance (ANOVA) was used to assess the data statistically. To examine the significance of changes in treatments, the least significant difference (LSD) at a 0.05 level of probability was utilized.

## Results

### Biochar characterization

RSB with pH 9.09 contained 0.52% total N, 0.37% total P and 9.2% total K (Table 1). The ACB had a pH value of 7.26 with 0.20% total N, 0.11% total P, and 0.85% total K. RSB contained a high concentration of K due to high K contents in rice straw used as a source for biochar production. It also contained a higher concentration of P compared to ACB. The RSB was observed to be more alkaline compared to ACB. The EC of RSB and ACB were measured to be 0.85 dS m^−1^ and 0.13 dS m^−1^, respectively.

### Ammonical nitrogen

**Figure 1:**
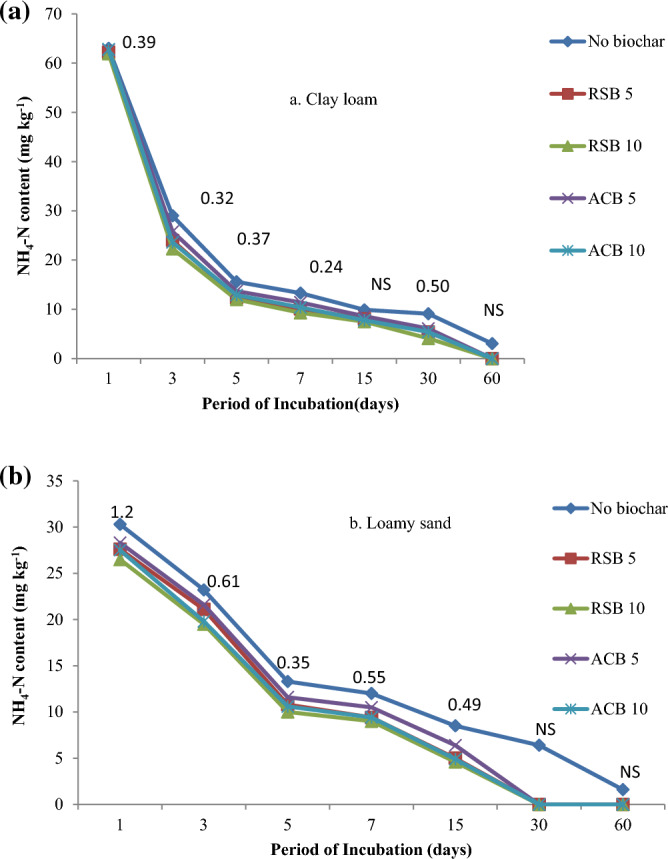
Interaction effect of type and rate of biochar on NH_4_-N content (mg kg^−1^) during a 60-day incubation period in (**a**) clay loam and (**b**) loamy sand soil, where 5 and 10 represents rate of biochar application @ 0.5 and 1.0% w/w basis.

At all sampling periods, except 15 and 60 days after incubation in clay loam and 30 and 60 days after incubation in loamy sand soil, a significant interaction of type and rate of biochar was detected (Fig. 1). Starting on day one and continuing until the end of the 60-day incubation, the NH_4_-N level was considerably lower in both soils supplemented with biochar compared to unamended soil (Fig. 1). Increasing the biochar rate from 5 to 10g kg^−1^ soil resulted in generally non-significant effect of NH_4_-N content on different sampling dates. In clay loam soil, NH_4_–N contents one day after incubation were similar (62.5 and 62.3 mg kg^−1^) with the mixing of (0.5 and 1%, w/w) biochar in soil and decreased gradually to 0.1 mg kg^−1^ at the end of the 60-day incubation period (Fig. 1a). The corresponding values of NH_4_-N for loamy sand soil were markedly lower (27.9 and 26.9 mg kg^−1^), which declined to 0.2 mg kg^−1^ at 30 days after incubation (Fig. 1b). Biochar type was not able to affect the NH_4_–N content. Biochars showed a greater effect on NH_4_–N content in loamy sand compared to clay loam soil. The concentration of NH_4_–N in the soil at 30 days after incubation was decreased to 8.68 and 6.62% of the concentration at day 1 after incubation when RSB was mixed with clay loam soil at the rate of (0.5 and 1.0%, w/w), respectively. In clay loam, the corresponding values of NH_4_-N concentration with the addition of ACB were 9.72 and 8.59%, respectively. The concentration of NH_4_-N at 30 days after incubation decreased more rapidly when RSB was applied compared to ACB, suggesting that RSB had a greater effect on increasing the nitrification rate in clay loam, while similar effects were observed at 15 days after incubation in loamy sand soil.

**Figure 2:**
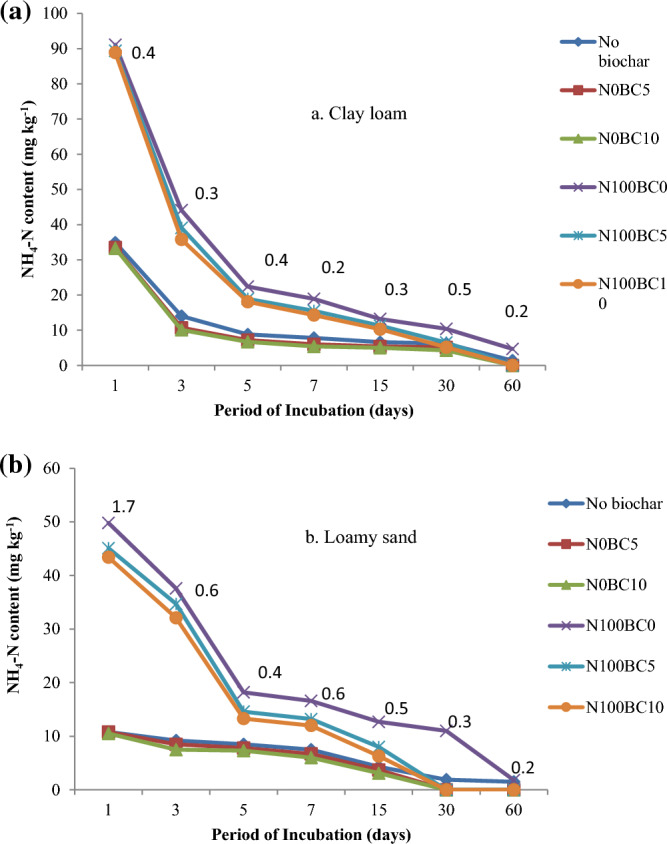
Interaction effect of different rates of biochar and N levels on NH_4_-N content (mg kg^−1^) during a 60-day incubation period in (**a**) clay loam and (**b**) loamy sand soil.

A significant interaction between N levels and rates of biochar (averaged across two types of biochar) was observed in NH_4_-N concentration in both the soil types on all the sampling dates (Fig. 2). The concentration of NH_4_-N was more in clay loam than in loamy sand soil due to higher initial content of NH_4_-N. As expected, the application of 100 mg urea-N kg^-1^ soil resulted in a significantly higher concentration of NH_4_-N compared to no-N control treatment on all the sampling dates. The NH_4_-N concentration in N-amended clay loam was significantly lower when biochar was added at 1% level as compared to 0 and 5% levels. However, no significant effect of biochar rate was observed in no-N treatment in clay loam (Fig. 2a). Generally, similar effects were observed in loamy sand soil (Fig. 2b). The NH_4_–N concentration in urea-amended soil decreased at a faster rate between 1 and 7 days of incubation compared to that between 7 to 15 days of incubation and thereafter, mainly due to nitrification. The decrease in NH_4_–N concentration in N-amended soil was significantly higher when 1% biochar was mixed with soil as compared to 0.5% level. At 30 and 60 days after incubation in N-supplemented loamy sand and clay loam, the concentration of NH_4_–N in loamy sand was reduced to a very low level (0.2 mg kg^−1^) with adding 0.5 or 1% biochar nitrate nitrogen.

**Figure 3:**
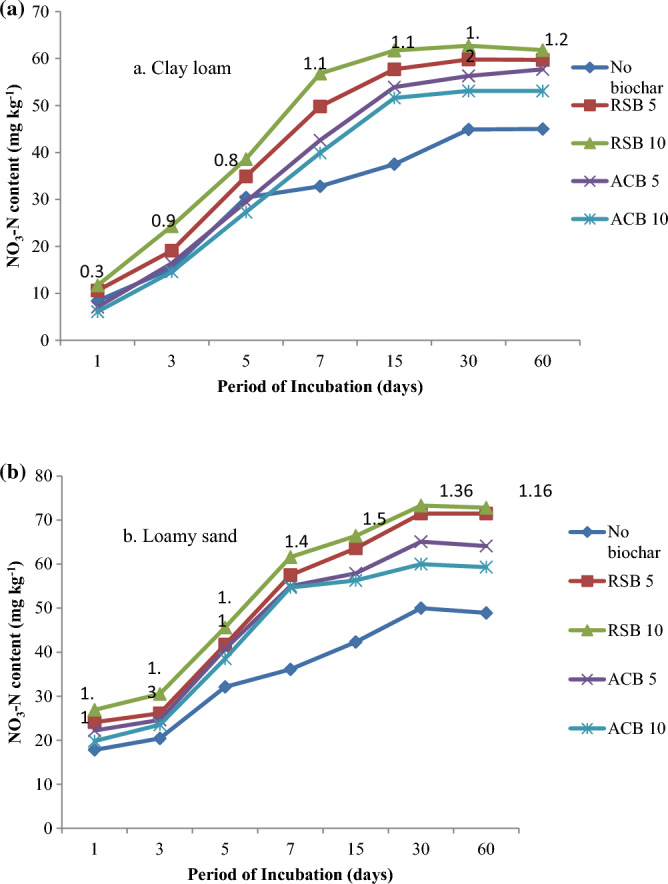
Interaction effect of type and rate of biochar on NO_3_-N content (mg kg^−1^) during a 60-day incubation period in (**a**) clay loam and (**b**) loamy sand soil.

Significant interaction effects of types and rates of biochar on NO_3_-N content were observed at all sampling periods in both soils. The NO_3_-N contents continued to increase throughout the 60-day incubation period in both soils due to the nitrification of applied N (Fig. 3a,b). Except for 60 days after incubation, all subsequent sampling dates in clay loam soil showed increased NO_3_-N content with a higher biochar rate. The contents of NO_3_-N in loamy sand soil increased with increasing biochar rate until 15 days after incubation. However, they decreased when 1% biochar was combined compared to 0.5 per cent biochar treatment at 30 and 60 days after incubation. On all sampling dates in both soils, RSB treated at 0.5 or 1.0% induced a considerably larger rise in NO_3_–N content than ACB. While the application of RSB at 1.0% level compared to 0.5% resulted in a significant increase in NO_3_–N content on all sampling dates, however, the reverse trend was observed in the case of ACB. Increasing the rate of ACB from 5 to 10 g kg^−1^ soil resulted in a significant decrease in NO_3_–N contents on all sampling dates in both soils. The NO_3_–N contents (averaged over biochar types) were 8.4, 8.0 and 8.9 mg kg^-1^ with the addition of 0, 0.5 and 1.0% biochar in clay loam soil one day after incubation. They increased to 44.9, 56.5 and 58.1 mg kg^-1^ 30 days after incubation, respectively. The corresponding values of NO_3_-N content in loamy sand were 7.8, 13.1 and 13.4 mg kg^−1^ with adding 0, 0.5 and 1.0% biochar at 1 day and increased to 50.2, 67.8 and 66.1 mg kg^−1^ at 30 days after incubation. The increase in the concentration of NO_3_–N at 15 days after incubation was 18.3 and 18.9% of the concentration observed one day after incubation with the addition of 0.5 and 1.0% RSB in clay loam soil. The corresponding increase in NO_3_-N concentration in loamy sand was 37.8 and 40.5%, respectively.

The effect of biochar addition in increasing NO_3_–N concentration was lower initially and then increased faster on or seven days until 60 days after incubation in both soils. This was primarily because loamy sand had a greater starting concentration of NO_3_–N than clay loam soil. There were no significant interaction effects of biochar rate and N-rate on NO_3_–N contents in both soils. As expected, the application of urea caused a significant increase in NO_3_-N contents at all the sampling dates in both soils (Table 3). The increase in NO_3_-N contents in soil treated with 100 mg urea-N kg^-1^ soil over No-N control was 33.8 mg and 51.0 mg kg^−1^ in clay loam and 24.2 mg and 38.2 mg kg^−1^ in loamy sand at 5 and 30 days after incubation, respectively (Table 3). The NO_3_–N contents in No-N control soil increased from 8.0 mg at 1 day to 27.4 mg kg^−1^ 30 days after incubation in clay loam and 10.6 mg to 38.7 mg kg^−1^ in loamy sand soil. Thus, an increase in NO_3_–N contents during the incubation is more in loamy sand compared to clay loam soil.

**Table 3:** Effect of N application on NO_3_-N (mg kg^−1^) accumulation (averaged over type and rates of biochar) at different sampling dates in clay loam and loamy sand soils.

Treatment	Incubation time (days)						
	1	3	5	7	15	30	60
Rate of N applied (mg kg^−1^)							
A. Clay loam							
0	8.0b	12.4a	14.9b	17.0b	26.0b	27.4b	27.8b
100	8.7a	22.6b	48.7a	68.0a	72.4a	78.4a	79.4a
B. Loamy sand							
0	10.6b	18.3b	26.3b	34.5b	36.0b	38.7b	38.8b
100	18.2a	30.2a	50.5a	65.8a	73.6a	76.9a	75.4a

Within a column, means followed by the same letter are not significantly different at a 0.05 probability level.

RSB resulted in significantly higher nitrification than ACB throughout the 60-day incubation period in both soils (Table 4). A significant increase in nitrification with biochar was observed up to mixing of 0.5%. There was no further increase in nitrification rate at the 1.0% level of biochar mixing 5 days after incubation and after that in both soils (Table 4). At 15 days after incubation, nitrification of applied N with no biochar treatment was 31.5%, which increased to 53.5% with 0.5% biochar, irrespective of the type of biochar in clay loam. Compared to clay loam soil, the nitrification rate of administered N was substantially slower during the first three days after incubation in loamy sand. However, the nitrification rate in loamy sand was substantially quicker than in clay loam for the remaining incubation duration. The nitrification rate in clay loam was 30 days, and 15 days in loamy sand soil. The nitrification in biochar amended treatment decreased markedly at 30 and 60 days compared to 15 days after incubation in loamy sand soil. The decrease in nitrification in loamy sand soil may be due to the loss of NO_3_–N or immobilization of NO_3_-N during incubation.

**Table 4:** Effect of type and rate of biochar application on nitrification (%) of applied urea-N.

Treatment	Incubation time (days)						
	1	3	5	7	15	30	60
Clay loam							
Type of biochar							
RSB	3.5a	12.3a	37.0a	55.4a	48.9.1a	53.2a	53.4a
ACB	0.6b	8.1b	31.5b	47.1b	44.2b	48.6b	49.8b
Rate of biochar (g kg^−1^)							
0	1.3a	10.2a	32.1b	35.0b	31.5b	45.5b	45.6b
5	1.7a	10.5a	35.4a	58.0a	53.5a	52.6a	54.3a
10	2.1a	9.9a	35.0a	60.8a	54.5a	54.9a	54.8a
Loamy sand							
Type of biochar							
RSB	5.3a	13.3a	26.3a	32.4a	40.7a	41.5a	33.4a
ACB	2.3b	10.5b	23.6b	29.3b	35.5b	34.9b	26.5b
Rate of biochar (g kg^−1^)							
0	4.4a	15.3a	20.0b	16.9b	26.9b	21.5b	29.6b
5	3.4a	10.5b	26.3a	38.7a	43.8a	31.4a	30.2a
10	3.5a	9.8b	28.4a	37.2a	43.5a	31.6a	30.0a

LSD 0.05 for interaction: NS.

RSB-Rice straw biochar, ACB-acacia biochar.

Within a column, means followed by the same letter are not significantly different at a 0.05 probability level.

### Available phosphorus

**Figure 4:**
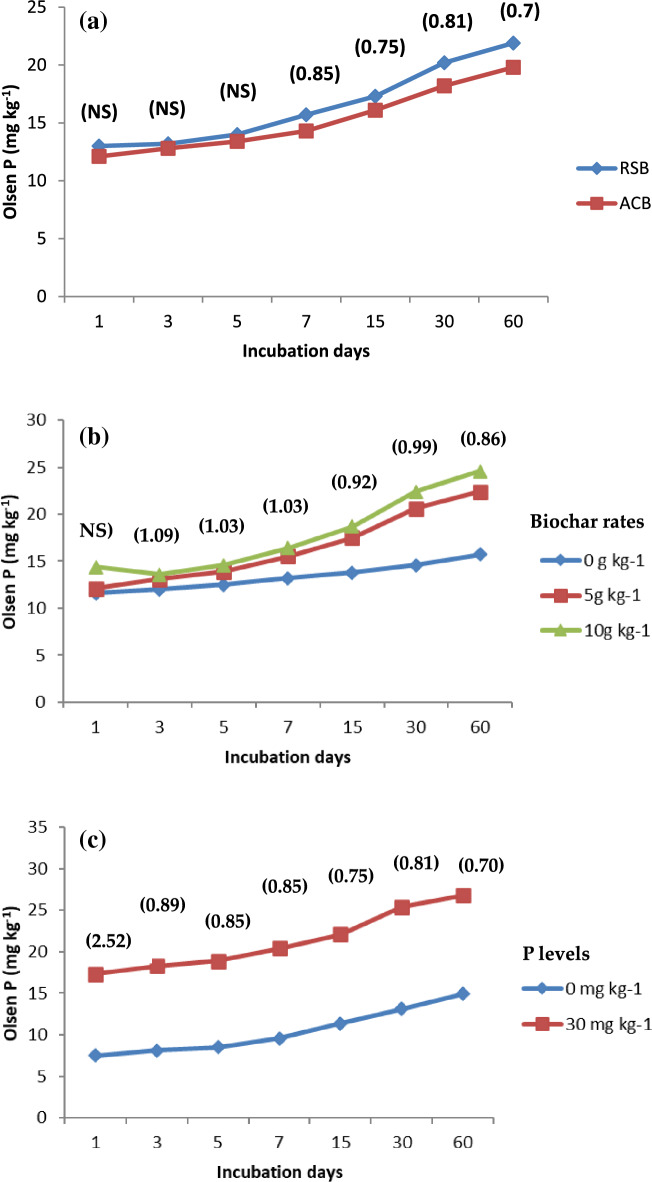
Effect of (**a**) biochar type (**b**) biochar rates and, (**c**) levels of P on Olsen P (mg kg^−1^) in Clay loam soil.

**Figure 5:**
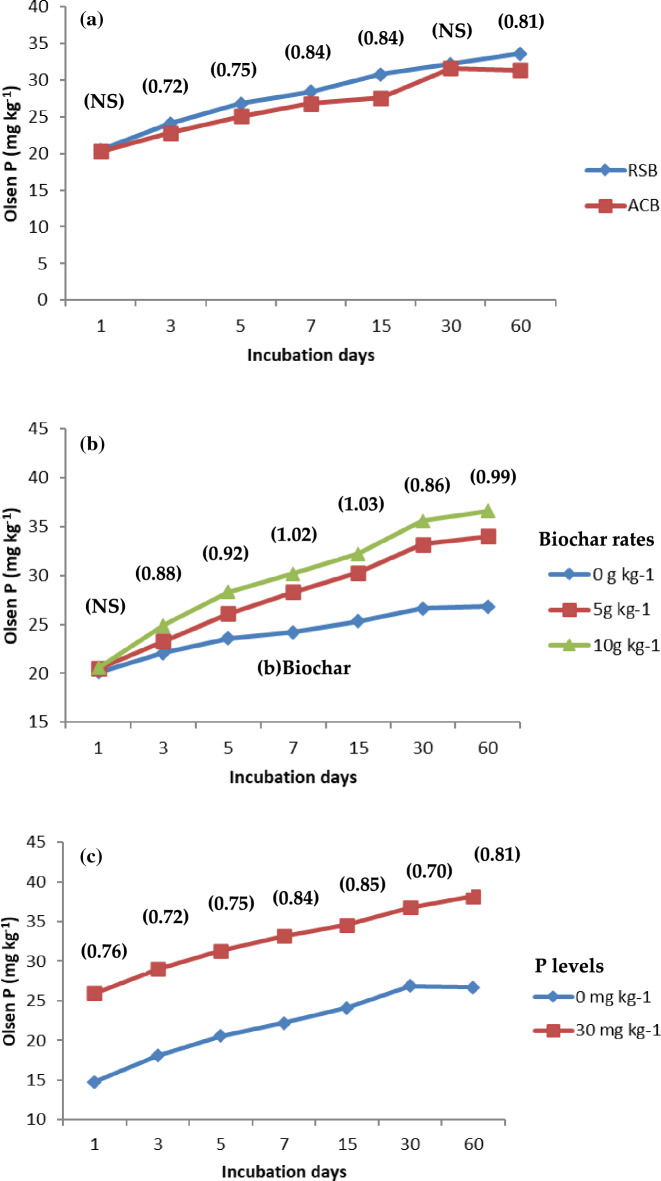
Effect of (**a**) biochar type (**b**) biochar rates and, (**c**) levels of P on Olsen P (mg kg^−1^) in Sandy loam soil.

RSB resulted in a significant increase in Olsen-P content in soil compared to ACB at 3 (in loamy sand) and 7 (clay loam soils) days after incubation (Figs. 4 and 5). At the end of the 60-day incubation period, Olsen-P remained consistently higher in both soils amended with RSB than the ACB. The increase in the mean concentration of Olsen-P (averaged over rates of biochar) at 60 days after incubation was 68.5 and 63.6% higher for RSB and ACB, respectively, compared to that at one day after incubation in the clay loam soil. The corresponding increase in Olsen-P in loamy sand soil was 58.0 and 55.7%. The addition of 0.5 and 1.0% biochar significantly increased (averaged over two types of biochar) Olsen-P content over No-P control during the entire period of incubation starting from 3 days after incubation. The addition of 1.0% biochar significantly increased P availability as compared to 0.5%. At 60 days after incubation, the increase in Olsen-P was 42.7 and 56.7% with the addition of 0.5 and 1.0% biochar (averaged over two types) compared to No-P control in clay loam soil and 26.9 and 36.6% in loamy sand soil, respectively. The application of 1.0% biochar significantly increased P availability as compared to the addition of 0.5% biochar in both soils. The rise in Olsen-P concentration in soil was greater in loamy sand soil than in clay loam soil.

### Principal component analysis (PCA)

Principal component analysis (PCA) of type and application rate of biochar on NH_4_–N content (mg kg^−1^) during a 60-day incubation period in clay loam and loamy sand soils are given in Supplementary Table S1 and Fig. S1 and application of different rates of biochar and N levels on NH4-N content (mg kg^−1^) during a 60-day incubation period in clay loam and loamy sand soil are given in Table S2 and Fig. S2.

## Discussion

The effectiveness of biochar on soil properties is a multifactor phenomenon and depends on biochar synthetic methodology, soil type, biochar levels and so on^26–28^. The biochar applications have been known to influence nutrient applications, and the results are optimum if the BCs are prepared under the best conditions. The present experiment had the biochars prepared at a moderate temperature (380 °C), at which they were expected to show the maximum cation exchange capacities (CEC)^29^. The augmented pyrolysis temperatures are reported for the decline in CEC as explained by the decrease of some functional groups, such as –COOH and –OH groups^30^, along with a decrease in aliphatic, amide, and aromatic amines^31^.

The applications of optimally produced BC herein resulted in significantly lower NH_4_–N in both soils amended with biochar compared to unamended soil starting at one day until the end of 60-day incubation (Fig. 1), on line with^32^^,^^33^. This is attributed to the functional groups, such as carboxyl and ketone groups, which play an important role in NH_4_^+^ retention owing to hydrogen bonding and electrostatic interaction. Additionally, BC application decreased the ammonia volatilization from the soil because of ammonium ions retention by increasing the soil cation exchange capacity^34,35^, resulting in a reduction of cumulative NH_4_^+^–N losses, which varied with biochar application in a dose-dependent manner^36^. Another report^37^ illustrated that biochar could adsorb NH_4_^+^-N predominantly through its high cation exchange capacity, resulting in a 15.2% reduction in cumulative NH_4_^+^-N losses. Amin^38^ quantitatively showed the kinetic evidence of lower ammonia volatilization with a higher half-life affecting total available nitrogen on BC application. In the present experiment, the NH_4_-N concentration in N-amended clay loam was significantly lower when biochar was added at 10 g kg^−1^ soil compared to 0 and 5 g kg^−1^ soil with no significant effect of biochar rate was observed in no-N treatment in clay loam (Fig. 2a).

The NO_3_-N contents continued to increase throughout the 60-day incubation period in both soils due to the nitrification of applied N (Fig. 3a,b) in the present experiment, as supported by Refs.^33^^,^^39^. The increase in the rate of ABC might have caused the immobilization of soil N due to a higher C/N ratio than RSB^27,28,40^. Contrarily, Dempster et al.^36^ reported a significant decrease in net nitrification by applying Eucalyptus biochar (wood biochar) with increasing biochar addition in coarse-textured soils. Nitrogen mineralization is usually more rapid in sandy soils than in loam or clay soils due to the physical protection of organic matter and microbial biomass by clay layers^41,42^.

Char materials are reported to influence soil P availability by altering P sorption capacity^43^. A significant augmentation in the Olsen-P content in soil with RSB was observed as compared to ACB at 3 (in loamy sand) and 7 (clay loam soils) days after incubation (Figs. 4 and 5). Then, its consistently higher contents in both the soils amended with RSB compared to the ACB at the end of the 60-day incubation period is well supported by the findings of^44,45^^,^^16^^,^^46^. Soil retained phosphorus in its particles in addition to biochar, leading to phosphorus supply enhancement and the reduction of environmental pollution^47,48^. These studies reported that the content of Olsen-P increased significantly with the application of maize stalk biochar compared to the control. The concentration of Olsen-P in soil increased further with the increase in the biochar application rate because of its higher potential for unlocking P^3^.

## Conclusions

The study concluded that adding 5 g rice straw biochar kg ^-1^ soil on a weight basis in low N soils can support the growth of crops by continuously supplying NO_3_-N in both soils by increasing nitrification significantly. It also offers an alternative for soils rich in P content for better bioavailability of P because of its higher potential for unlocking P. ACB shows the most potent effect for externally supplied P in a clay loam system by mixing 5 g ACB kg^−1^ soil. It can help farmers to apply appropriate doses of locally available biochar as a soil amendment.

### Ethics

All the authors abide by the IUCN Policy Statement on Research Involving Species at Risk of Extinction and the Convention on the Trade in Endangered Species of Wild Fauna and Flora.

### Supplementary Information

Supplementary Information.

## Data Availability

The datasets used and/or analysed during the current study are available from the corresponding author upon reasonable request.
