# Supplementary material for: Biochar influences nitrogen and phosphorus dynamics in two texturally different soils
Source: Sci Rep. 2024 Mar 19;14:6533. doi: 10.1038/s41598-024-55527-2 (PMC10951405; doi:10.1038/s41598-024-55527-2)
Supplement: Supplementary file 1 — Supplementary Information. [file 41598_2024_55527_MOESM1_ESM.docx]

**Supplementary Information**

**Biochar influences nitrogen and phosphorus dynamics in two texturally different soils**

Rajeev Kumar Gupta ^1,^*, Monika Vashisht ^1^, R. K. Naresh ^2^, Nitish Dhingra ^3^, Mehra S. Sidhu ^3^, P. K. Singh ^4^, Neeraj Rani ^5^, Nadhir Al-Ansari ^6,^*, Abed Alataway ^7^, Ahmed Z. Dewidar ^7,8^ and Mohamed A. Mattar ^7,8,^*

^1^ School of Agriculture, Lovely Professional University, Jalandhar 144001, Punjab, India; [mvahisht3669@gmail.com](mailto:mvahisht3669@gmail.com) (M.V.)

^2^ Department of Agronomy, Sardar Vallabhbhai Patel University of Agriculture & Technology, Meerut-250110, U.P., India; [r.knaresh@yahoo.com](mailto:r.knaresh@yahoo.com) (R.K.N.)

^3^ Electron Microscopy & Nanoscience Laboratory, Department of Soil Science, Punjab Agricultural University, Ludhiana 141004, Punjab, India; [nitishdhingra@pau.edu](mailto:nitishdhingra@pau.edu) (N.D.); [sidhums@pau.edu](mailto:sidhums@pau.edu) (M.S.S.)

^4^ Director Extension, Sardar Vallabhbhai Patel University of Agriculture & Technology, Meerut-250110, U.P., India; [spraveen681@gmail.com](mailto:spraveen681@gmail.com) (P.K.S.)

^5^ School of Organic Farming Punjab Agricultural University Ludhiana-141004, India; [neerajsoil@pau.edu](mailto:neerajsoil@pau.edu) (N.R.)

^6^ Department of Civil, Environmental and Natural Resources Engineering, Lulea University of Technology, 97187 Lulea, Sweden

^7^ Prince Sultan Bin Abdulaziz International Prize for Water Chair, Prince Sultan Institute for Environmental, Water and Desert Research, King Saud University, P.O. Box 2454, Riyadh 11451, Saudi Arabia; aalataway@ksu.edu.sa (A.A.), [adewidar@ksu.edu.sa](mailto:adewidar@ksu.edu.sa) (A.Z.A.)

^8^ Department of Agricultural Engineering, College of Food and Agriculture Sciences, King Saud University, P.O. Box 2460, Riyadh 11451, Saudi Arabia; [mmattar@ksu.edu.sa](mailto:mmattar@ksu.edu.sa), <https://orcid.org/0000-0002-7506-3036> (M.A.M.)

*****Correspondence: [rajeev.30662@lpu.co.in](mailto:rajeev.30662@lpu.co.in), [rkg1103@pau.edu](mailto:rkg1103@pau.edu) (R.K.G.), nadhir.alansari@lttu.se (N.A.-A.), [mmattar@ksu.edu.sa](mailto:mmattar@ksu.edu.sa) (M.A.M.).

**Section 1: Principal component analysis (PCA) of type and application rate of biochar on NH_4_-N content (mg kg^-1^) during a 60-day incubation period in clay loam and loamy sand soils**

Principal Component Analysis (PCA) is also a projection method whose primary objective is to explain the relation among the large data sets and convert large data into smaller and more informative components. A PCA approach was employed to detect the relation between different types of biochar, namely Rice Straw Biochar (RSB) and Acacia Wood Biochar (ACB) and the rate of biochar treatment and incubation period for the content of NH_4_-N in clay loam (CL) and Loamy Sand (LS) soils. In total, 10 principal components were considered for initial analysis; no biochar or biochar was applied at the rate of 0.5, 1.0 % w/w for the incubation period of 1,3,5,7,15,30 and 60 days after treatment. In the correlation matrix, shown in Table S1, the variables are highly correlated except for one at the incubation period 60, where most values are greater than 0.3.

**Table S1.** Correlation Matrix of incubation period about the NH_4_-N content and type of biochar and rate of biochar applied in clay loam and sandy loam soils.

| Incubation Period | 1 | 3 | 5 | 7 | 15 | 30 | 60 |
| --- | --- | --- | --- | --- | --- | --- | --- |
| 1 | 1 | 0.74175 | 0.68162 | 0.36033 | 0.75872 | 0.76879 | 0.17573 |
| 3 | 0.74175 | 1 | 0.97132 | 0.86678 | 0.92274 | 0.90777 | 0.69693 |
| 5 | 0.68162 | 0.97132 | 1 | 0.92368 | 0.96011 | 0.93988 | 0.74839 |
| 7 | 0.36033 | 0.86678 | 0.92368 | 1 | 0.83379 | 0.80119 | 0.85494 |
| 15 | 0.75872 | 0.92274 | 0.96011 | 0.83379 | 1 | 0.96479 | 0.62717 |
| 30 | 0.76879 | 0.90777 | 0.93988 | 0.80119 | 0.96479 | 1 | 0.66555 |
| 60 | 0.17573 | 0.69693 | 0.74839 | 0.85494 | 0.62717 | 0.66555 | 1 |

[Figure S1 (a)](https://www.mdpi.com/2073-4395/13/1/113#fig_body_display_agronomy-13-00113-f006) illustrate the screen plot where the elbow point exists at principal component 4. However, only the first two components have values higher than 1. Thus, as shown in Figure [S1 (b)](https://www.mdpi.com/2073-4395/13/1/113#app1-agronomy-13-00113), the first two principal components (PCs) that accounted for ~95 % of the total variance were extracted from the original datasets and considered for further analysis. Most variables were positioned in PC1, suggesting it contained more useful information than the other PCs. According to the loading plot, the first principal component has significant positive associations with the incubation period, so this component primarily measures the NH_4_-N content in CL and LS soils for up to 60 days or more in clay loam and loamy sand soils. According to the biplot (Fig S1 (b)), except for RSB and ACB applied @ 0.5 and 1 % in loamy sand soils, which fell along the negative axis of PC1, the other 6 parameters were weighted on the positive axis. A significant interaction between N levels and rates of biochar (averaged across two types of biochar) was observed in NH_4_-N concentration in both the soil types on all the incubation days. The concentration of NH_4_-N was higher in clay loam than in loamy sand soil due to the higher initial content of NH_4_-N.

 ****

Fig S1. (a) Screen plot of eigenvalues vs principal component number. The black arrow represents the elbow point that signifies the number of primary principal components (b) Biplot of first (PC1) and second (PC2) principal components of 10 evaluated traits with different biochar treatments in clay loam (CL) and loamy sand soils for the incubation period of 60 days.

**Section 2. Principal component analysis (PCA) of the application of different rates of biochar and N levels on NH_4_-N content (mg kg^-1^) during a 60-day incubation period in clay loam and loamy sand soil**

A PCA approach was employed to detect the relation between different rates of biochar (0, 0.5 and 1.0 % w/w) treatment, Nitrogen applied @ 0 and 100 mg/kg and incubation period of 1, 3, 5, 7, 15, 30 and 60 days for the content of NH4- N in clay loam (CL) and Loamy Sand (LS) soils. Herein, 12 principal components were considered for initial analysis, including no biochar, and biochar applied at the 0.5 and 1.0 % w/w rate in both soils. The nitrogen application effect on NH4-N as a function of the incubation period was also evaluated. Table S2 illustrates the correlation matrix of the variables, which are highly correlated except for a few at the incubation period of 60, while most values are greater than 0.3.

**Table S2.** Correlation Matrix of incubation period about the NH_4_-N content and rate of biochar and nitrogen applied in clay loam and sandy loam soils.

| Incubation Period | 1 | 3 | 5 | 7 | 15 | 30 | 60 |
| --- | --- | --- | --- | --- | --- | --- | --- |
| 1 | 1 | 0.86929 | 0.88952 | 0.83824 | 0.88009 | 0.61695 | 0.33784 |
| 3 | 0.86929 | 1 | 0.97003 | 0.97603 | 0.91572 | 0.50507 | 0.37938 |
| 5 | 0.88952 | 0.97003 | 1 | 0.99175 | 0.94856 | 0.59841 | 0.50303 |
| 7 | 0.83824 | 0.97603 | 0.99175 | 1 | 0.9459 | 0.59036 | 0.52262 |
| 15 | 0.88009 | 0.91572 | 0.94856 | 0.9459 | 1 | 0.79657 | 0.54335 |
| 30 | 0.61695 | 0.50507 | 0.59841 | 0.59036 | 0.79657 | 1 | 0.65764 |
| 60 | 0.33784 | 0.37938 | 0.50303 | 0.52262 | 0.54335 | 0.65764 | 1 |

[Figure S2 (a)](https://www.mdpi.com/2073-4395/13/1/113#fig_body_display_agronomy-13-00113-f006) illustrate the screen plot where the elbow point exists at principal component 3. However, only the first two components have eigenvalues higher than 1. Thus, as shown in Figure [S1 (b)](https://www.mdpi.com/2073-4395/13/1/113#app1-agronomy-13-00113), the first two principal components (PCs) that accounted for ~91 % of the total variance were extracted from the original datasets and considered for further analysis. A linear combination of the original predictor variables yields the first principal component, which represents the highest variance in the data set. It establishes which way the data is most variable. Each principal component gathers greater information, and more variability is caught in the initial component. Any other component did not exceed the variability of the first principal component. The variables were positioned on the positive side of PC1, suggesting that it contained more useful information than the other PCs. According to the loading plot, the first principal component has significant positive associations with the incubation period, so this component primarily measures the higher content of NH4-N in simultaneously applied biochar (0.5, 1.0 %) and Nitrogen (100 mg/kg) in clay loam and loamy sand soils than only biochar applied @ 0.5 and 1.0 % w.r.t. control where no nitrogen and biochar was applied.

According to the biplot (Fig S2 (b)), the biochar applied @ 0.5 and 1.0 %, as well as control (no biochar and no Nitrogen) in clay loam and loamy sand soils fell along the negative axis of PC1, while the other 6 parameters (Bochar applied @ 0.5 and 1.0 % and N @ 100 mg/kg) were weighted on the positive axis. A significant interaction between N levels and rates of biochar was observed in NH_4_-N concentration in both soil types on all the incubation days. The concentration of NH_4_-N was higher in clay loam than in loamy sand soil due to the higher initial content of NH_4_-N. As anticipated, on every measurement day, the application of 100 mg urea-N kg^-1^ soil led to a noticeably greater concentration of NH_4_-N compared to the no-N control treatment.

Fig S2. (a) Screen plot of eigenvalues vs principal component number. The black arrow represents the elbow point that signifies the number of primary principal components (b) Biplot of first (PC1) and second (PC2) principal components of 12 evaluated traits with different biochar (0,0.5,1.0%) and nitrogen (0, 100 mg/kg) treatments in clay loam (CL) and loamy sand soils for the incubation period of 60 days.
